# The *raspberry* Gene Is Involved in the Regulation of the Cellular Immune Response in *Drosophila melanogaster*

**DOI:** 10.1371/journal.pone.0150910

**Published:** 2016-03-04

**Authors:** Beáta Kari, Gábor Csordás, Viktor Honti, Gyöngyi Cinege, Michael J. Williams, István Andó, Éva Kurucz

**Affiliations:** 1 Immunology Unit, Institute of Genetics, Biological Research Centre of the Hungarian Academy of Sciences, Szeged, Hungary; 2 Functional Pharmacology, Department of Neuroscience, Uppsala University, Uppsala, Sweden; Institute of Plant Physiology and Ecology, CHINA

## Abstract

*Drosophila* is an extremely useful model organism for understanding how innate immune mechanisms defend against microbes and parasitoids. Large foreign objects trigger a potent cellular immune response in *Drosophila* larva. In the case of endoparasitoid wasp eggs, this response includes hemocyte proliferation, lamellocyte differentiation and eventual encapsulation of the egg. The encapsulation reaction involves the attachment and spreading of hemocytes around the egg, which requires cytoskeletal rearrangements, changes in adhesion properties and cell shape, as well as melanization of the capsule. Guanine nucleotide metabolism has an essential role in the regulation of pathways necessary for this encapsulation response. Here, we show that the *Drosophila* inosine 5'-monophosphate dehydrogenase (IMPDH), encoded by *raspberry* (*ras*), is centrally important for a proper cellular immune response against eggs from the parasitoid wasp *Leptopilina boulardi*. Notably, hemocyte attachment to the egg and subsequent melanization of the capsule are deficient in hypomorphic *ras* mutant larvae, which results in a compromised cellular immune response and increased survival of the parasitoid.

## Introduction

Multicellular organisms have evolved diverse defense mechanisms against pathogenic microorganisms and parasites. Cellular encapsulation of invading parasites and endogenous tumors is a phylogenetically conserved mechanism of the innate immune response. Granuloma formation in vertebrates [1, 2] and capsule formation against endoparasitoid wasps in insects [3, 4] represent special forms of the cellular immune response. Furthermore, throughout the animal kingdom, these responses involve phylogenetically conserved signaling molecules and modules [5], as well as structures showing remarkable similarities, most likely as a result of convergent evolution of interactions between hosts and parasites [6]. In recent years, *Drosophila melanogaster* has revealed itself to be an excellent model for the investigation of granuloma formation and the encapsulation reaction.

The cellular immune response of *D*. *melanogaster* is confronted with a wide array of pathogenic microorganisms and parasites. Prokaryotes are ingested by phagocytic hemocytes (blood cells), the plasmatocytes [7], while large foreign objects, such as eggs from endoparasitoid wasps are encapsulated by hemocytes and destroyed within melanotic capsules [3]. The encapsulation response has a well defined order. First plasmatocytes attach and spread onto the wasp egg, after which they form septate junctions and enclose the egg in a capsule [8]. This is followed by the appearance of a specialized cell type, the lamellocyte, which differentiates in the sessile tissue, in the lymph gland and in the circulation [9, 10, 11]. These large flat cells bind to the egg, and eventually form a multilayered capsule. The final step in the encapsulation reaction is melanization of the capsule, which is accompanied by formation of potentially toxic quinones and free oxygen radicals [12, 13, 14]. This reaction is catalyzed by prophenoloxidase 3 (PPO3) expressed by lamellocytes [15].

To circumvent this cellular immune response, parasitoid wasps have evolved cytotoxic components or virus particles that are injected during oviposition [16]. In the case of successful encapsulation, reaction the fly survives, conversely, if the larva is immune deficient, or the wasp is able to inhibit the encapsulation response, the fly dies [17].

Guanine nucleotides have an evolutionary conserved role in the regulation of cell proliferation, differentiation, apoptosis, and are essential for cellular signaling and trafficking. Rho-family small GTPases (i.e. Rho, Rac1, Rac2 and Cdc42) are key regulators of the encapsulation process, operating in cytoskeletal rearrangements, lamellipodia and filopodia formation, cell shape changes and migration [18, 19, 20, 21, 22]. In the Rac2 mutant, due to defective filopodia formation, the abnormal spreading of plasmatocytes and lamellocytes results in an improper encapsulation reaction [20]. Furthermore, *Drosophila* small GTPases and other factors (i.e. the JNK homolog Basket, the dJNK kinase hemipterous, the TNF homolog Eiger), are involved in the release of prophenoloxidase from crystal cells [23].

The *Drosophila raspberry* gene encodes an inosine monophosphate dehydrogenase enzyme (IMPDH), which catalyzes the rate-limiting step of *de novo* synthesis of guanine nucleotides and thus regulates the GTP pool [24, 25]. It is a highly conserved essential enzyme found in all eukaryotes, as well as in most prokaryotes, and catalyzes the NAD^+^-dependent oxidation of inosine monophosphate (IMP) to xanthosine monophosphate (XMP). Recently, it was shown that IMPDH acts as a DNA binding transcriptional repressor attenuating the expression of the cell cycle-dependent transcription factor E2f, a key driver of cell proliferation. The catalytic activity of IMPDH is not required for sequence-specific DNA binding. As a nucleotide biosynthetic enzyme and transcription factor, IMPDH maintains the balance between metabolic state and cell proliferation [26]. Due to its crucial role, IMPDH is a major drug target for immunosuppressive, antiviral and anticancer therapy [24, 27, 28, 29].

In *Drosophila*, Raspberry is involved in Rho GTPase mediated cytokinesis [30], and in the phagocytosis of *Escherichia coli*, *Candida*. *albicans* in S2 cells [31]. Overexpression of *raspberry* in hemocytes causes plasmatocyte accumulation along the dorsal vessel, possibly due to changes in their adherence or migratory properties [32]. Here, we report that the *Drosophila raspberry* gene is involved in regulating the encapsulation reaction by influencing hemocyte adhesion and melanization of the capsule, as inhibition of *raspberry* leads to encapsulation defects and higher survival rates of the wasp *Leptopilina boulardi*.

## Materials and Methods

### *Drosophila* stocks

The *Drosophila* stocks *Oregon-R*, *w*^*1118*^, *ras*^*2*^, *y*^*1*^ sc* *v*^*1*^*; P{TRIP*.*HMC03250} attP2*, *y*^*1*^ *v*^*1*^*; P{TRIP*.*JF01446} attP2*, *Rac2*^*Δ*^ were obtained from the Bloomington Drosophila Stock Center (Bloomington, Indiana, USA). The following driver lines were used: *Hemese-Gal4 UAS-GFPnls* (*He-Gal4*) [33] and *msnF9mo-Gal4* [34, 35]. The flies were kept on standard cornmeal-yeast diet at 25°C.

### Antibodies

The P1a and P1b antibodies recognize the NimC1 molecule on plasmatocytes, the L1a, L1b and L1c antibodies [36] react with the Atilla molecule on lamellocytes [37, 38]. The antibodies were used as neat in the form of hybridoma tissue culture supernatants. The secondary antibody was goat anti-mouse CF-568 (Sigma-Aldrich), used at 1:1000 dilution.

### Immune induction with parasitoid wasp

The cell-mediated immune response was induced by using the parasitoid wasp *Leptopilina boulardi* strain G486. 72 h old *Drosophila* larvae (n = 50) were exposed to 8 female wasps and incubated for 2h at 25°C. After wasp infestation, the wasps were removed, and larvae were kept at 25°C or at 29°C.

### Eclosion of the *D*. *melanogaster* and *L*. *boulardi* adults after parasitization and statistics

Forty-eight hours after parasitization the *Drosophila* larvae were collected and washed in *Drosophila* Ringer’s solution (7.5 g NaCl, 0.35 g KCl, 0.21 g CaCl_2_, in 1000 ml dH_2_O, pH 7.0) and viewed for encapsulated and melanized wasp eggs or a small melanized black spot under a stereomicroscope. The parasitized larvae were transferred into vials containing standard fly food. The pupae were counted and then monitored for eclosing flies or wasps.

After 2 hours immune induction, the *raspberry* RNAi knockdown larvae were transferred to standard fly food and kept at 25°C or 29°C for 5 days respectively.

The experiments were repeated at least three times, summing up at least 100 flies/genotype. For the statistical analysis the Student’s *t*-test was used; the *p*-values of <0.05 were considered as significant.

### Encapsulation assay

The larvae were dissected on 12-spot microscope slides (SM-11, Hendley Essex) 48 or 72h after parasitization. The number of the live, partially encapsulated and melanized or fully encapsulated and melanized wasp larvae in each individual *Drosophila* larva were counted.

### Immunostaining of the encapsulated wasp eggs

Forty-eight and 72 hours after wasp infestation the larvae were dissected in multiwell glass plate, in Schneider’s medium containing 5% fetal bovine serum (FBS) and 0.003% 1-phenyl-2-thiourea (PTU) (Sigma-Aldrich), washed with PBS (137 mM NaCl, 2.7 mM KCl, 6.7 mM Na_2_HPO_4_, 1.5 mM, KH_2_PO_4_, pH 7.2), fixed in PBS containing 2% paraformaldehyde and washed three times in PBS for 5 min. The samples were blocked with PBS containing 0.1% bovine serum albumin (PBS-BSA) for 20 min and incubated overnight with the corresponding primary antibody. After washing three times with PBS, the CF-568 secondary antibody was added (diluted in PBS-BSA) and incubated for 45 min. The nuclei were stained with DAPI (Sigma-Aldrich). The samples were washed three times in PBS for 5 min each, mounted in Flouromount G (SouthernBiotech) and analyzed with a Zeiss Axioscope 2 MOT fluorescence microscope or a Leica confocal LSM.

### Hemocyte collection, counting and statistics

Third instar wandering larvae were bled into 30 μl *Drosophila* Ringer’s solution containing PTU by ripping the cuticle with two fine forceps. Hemocytes were counted from at least 8 larvae of each genotype in Bürker chamber. For the statistical analysis of total hemocyte number the Student’s *t*-test was used; the *p*-values of <0.05 were considered as significant.

### Immunostaining of circulating hemocytes

Larvae were dissected on 12-spot microscope slides in Schneider’s media containing 5% FBS and PTU, at the indicated time points. Hemocytes were incubated on microscope slides to adhere for 1 hour in humid chambers in Schneider’s medium containing 5% FBS, then fixed in 2% paraformaldehyde containing PBS. The samples were washed three times in PBS for 5 min and blocked with PBS-BSA for 20 min, then incubated 1 hour with the primary antibody. After washing three times with PBS, the secondary anti-mouse CF-568 antibody (diluted in PBS-BSA) was added and incubated for 45 min. The nuclei were stained with DAPI. The samples were washed three times in PBS for 5 min each, then mounted in Flouromount G and analyzed with Zeiss Axioscope 2 MOT fluorescence microscope.

### Examination of pseudopod-like cytoplasmic extensions in hemocytes

Twenty-four hours after infestation with *L*. *boulardi*, six larvae were collected from each group and bled into Schneider’s media containing 5% FBS and PTU. Hemocytes were incubated on 12-spot microscope slides for 1h, fixed in PBS containing 2% paraformaldehyde and washed three times in PBS for 5 min. The samples were blocked with 0.1% BSA and 0.01% Triton X-100 containing PBS for 20 min. The actin cytoskeleton was stained with phalloidin conjugated Atto Rho6G (Sigma-Aldrich, 1:1000 final dilution in PBS-BSA) for 45 min, then washed three times in PBS for 5 min each. The nuclei were stained with DAPI. The samples were mounted in Flouromount G (SouthernBiotech) and analyzed with a Zeiss Axioscope 2 MOT fluorescence microscope and the proportion of filopodia promoting hemocytes was established. Three independent biological samples were analyzed. The Student’s *t*-test was used to calculate the significant difference from the control samples. The *p*-values of <0.05 were considered significant.

## Results

### The *ras*^*2*^ allele affects the survival rate of the *Drosophila* larvae after infestation

**Fig 1:**
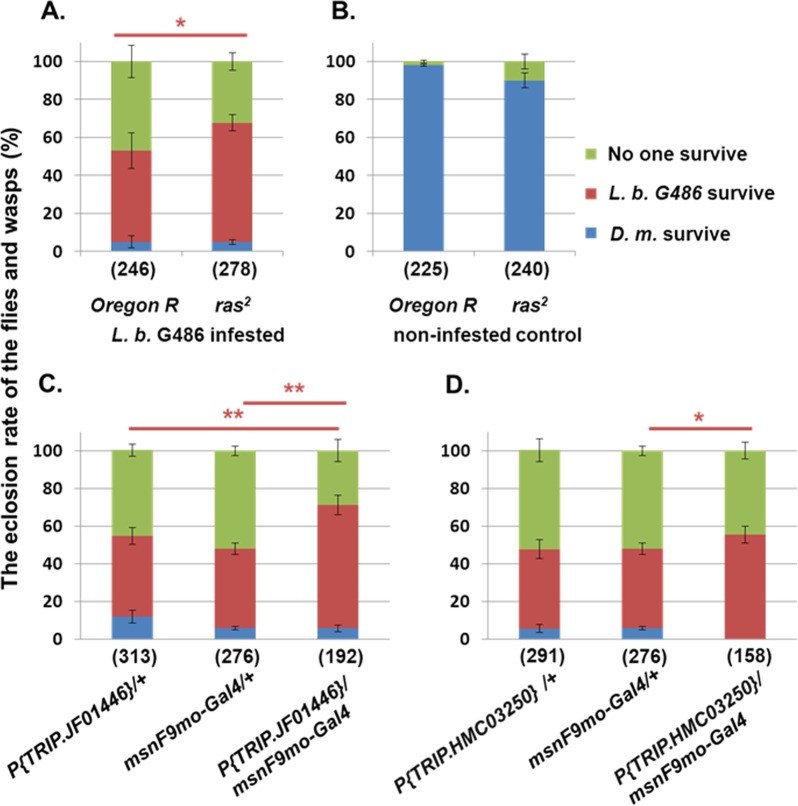
The eclosion of *D*. *melanogaster* versus *L*. *boulardi* G486 in *ras*^*2*^ mutant and after RNAi silencing. (A) The eclosion of flies (blue) and wasps (brown) from the *ras*^*2*^ mutant or from the *Oregon-R* after parasitization and from the (B) non-infested control. (C, D) Number of hatching flies from the *L*. *boulardi* G486 infested progenies (*msnF9mo-Gal4*>*P{TRIP*.*JF01446}* and *msnF9mo-Gal4*>*P{TRIP*.*HMC03250} attP2}*) and parental lines (*P{TRIP*.*JF01446}/+*, *P{TRIP>HMC03250/+* and *msnF9mo/Gal4/+*). The numbers in parenthesis indicate the number of the examined *D*. *melanogaster* pupae. The error bars indicate the standard error of the mean. *p<0.05, **p<0.01.

We determined the rate of survival of the *ras*^*2*^ mutant after *L*. *boulardi* G486 parasitization in the hypomorphic, fully viable *raspberry* allele in *D*. *melanogaster—*as the *ras* null mutants are lethal [39, 40]. We found that the number of the wasps eclosed from the *ras*^*2*^ mutant was significantly higher (*p*<0.05) from the *ras*^*2*^ pupae (Fig 1A, red column) than from the *Oregon-R* control. The number of *ras*^*2*^ pupae from which neither flies nor wasps emerged was significantly lower compared to wild type controls (Fig 1A, green column). We observed that the number of hatching flies was relatively low both in the mutant and the control (Fig 1A, blue column). In the non-infested *Oregon-R* and *ras*^*2*^, there was no significant difference in the eclosion rate (Fig 1B).

We also investigated the survival of fly versus wasp after depletion the *raspberry* specific mRNA transcript in blood cells. We used the *msnF9mo-Gal4* [34, 35] driver active in lamellocytes and lamellocyte precursors and the *Hemese-Gal4* (*He-Gal4*) [32] driver, which is active in 80% of hemocytes including plasmatocytes and lamellocytes to silence *ras* by two independent *ras* RNAi constructs (*y*^*1*^ sc* *v*^*1*^*; P{TRIP*.*HMC03250} attP2* and *y*^*1*^ *v*^*1*^*; P{TRIP*.*JF01446} attP2*) at 25°C (S1 and S2 Figs) and 29°C. (Fig 1C and 1D, S3 Fig). We monitored the eclosion of flies or wasps and found that significantly more wasps hatched from the *msnF9mo-Gal4*>*P{TRIP*.*JF01446}* progeny compared to the parental lines (*p* < 0.01) (Fig 1C). In the case of the other *raspberry* RNAi line (*y*^*1*^ sc* *v*^*1*^*; P{TRIP*.*HMC03250} attP2}*), the difference was also significant compared to the *msnF9mo-Gal4*/+ (*p* > 0.05) (Fig 1D). Similar results were obtained at 25°C using the *msnF9mo-Gal4* driver (S1 Fig) or by using the *He-Gal4* driver line at 25°C and 29°C (S2 and S3 Figs).

### *raspberry* is involved in encapsulation and melanization reactions

**Fig 2:**
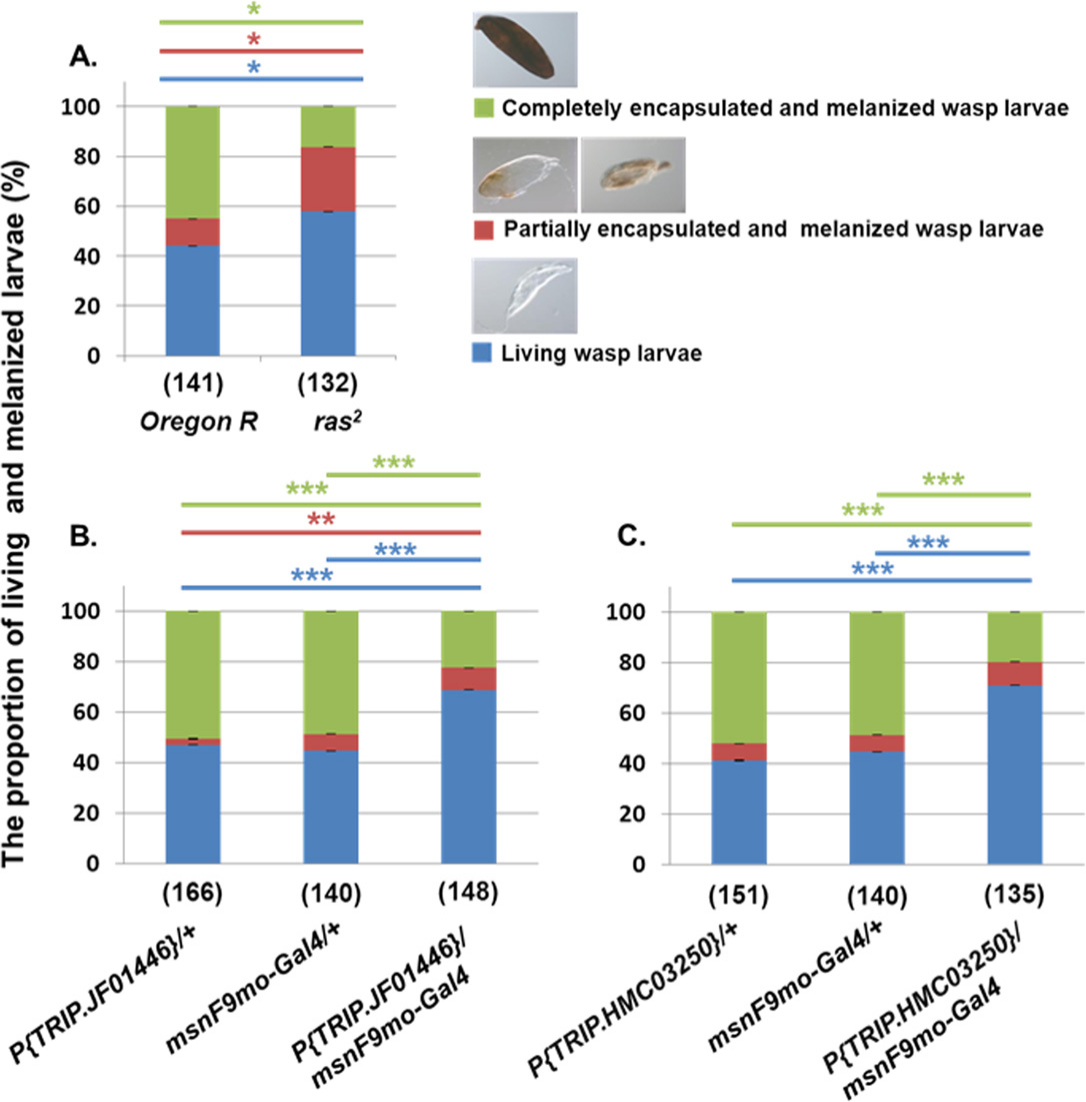
The phenotype and proportion of living wasp larvae, partially or completely encapsulated and melanized wasp eggs in *ras*^*2*^ mutant or after RNAi silencing. (A) The phenotypic categories of *L*. *boulardi* G486 larvae and capsules in *D*. *melanogaster* larvae after parasitization. Proportion of living wasp larvae (blue) and partially (red) or completely melanized (green) capsules in the *ras*^*2*^ mutant or in the *Oregon-R* control (B, C) and from the parental lines (*P{TRIP*.*JF01446}/+*, *P{TRIP>HMC03250/+* and *msnF9mo/Gal4/+*) and progenies (*P{TRIP*.*JF01446}>msnF(mo/Gal4* and *P{TRIP>HMC03250>msnF9mo/Gal4* 72h after the *L*. *boulardi* G486 infestation. The numbers in parenthesis indicate the number of the examined *Drosophila* larvae. The error bars indicate the standard error of the mean.*p<0.05, **p<0.01, ***p<0.001.

We investigated the phenotype and number of capsules in *D*. *melanogaster* after parasitization with *L*. *boulardi* G486. We counted non-encapsulated living wasp larvae, partially encapsulated and melanized or completely melanized wasp eggs (Fig 2A) in the hemocoel of the *ras*^*2*^ and *Oregon-R* larvae.

Seventy-two hours after oviposition, we counted the total number of parasitoids in the *ras*^*2*^ mutant and in the *Oregon-R* larvae, and we found no significant difference in the number of parasitoids, which indicates that *L*. *boulardi* G486 has no preference for depositing eggs into the *ras*^*2*^ or the *Oregon-R* larvae. At the same time, we detected significantly higher number of living or partially encapsulated and melanized wasp larvae and significantly lower number of completely melanized eggs in the *ras*^*2*^ mutant compared to the *Oregon-R* control after 48h (S4 Fig) or 72h following oviposition (Fig 2A). Similarly, using two different *ras* RNAi constructs driven by the *msnF9mo-Gal4* driver, we detected a significantly higher frequency of living wasp larvae in the progenies than in the parental lines 48h (S5 Fig) or 72h (Fig 2B and 2C) after oviposition. We obtained similar results using the hemocyte specific *He-Gal4* driver to knockdown *ras* expression (S6 and S7 Figs).

### Incomplete lamellocyte adherence to the capsule in the *ras*^*2*^ mutant

**Fig 3:**
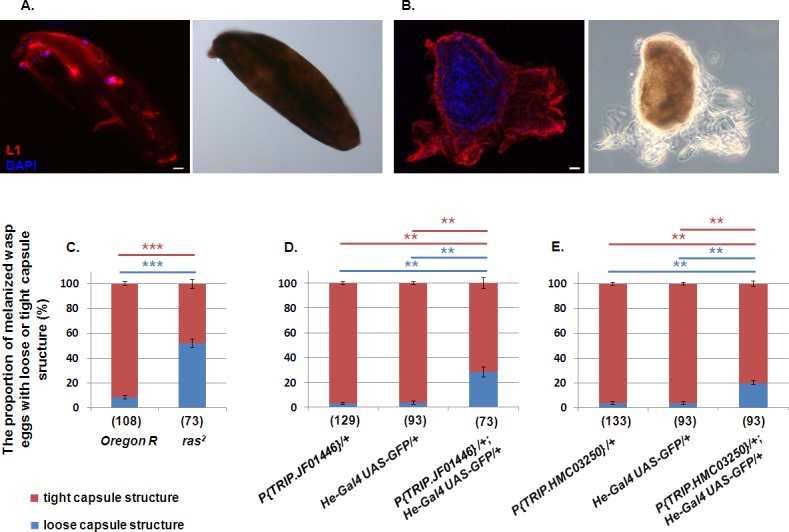
Attachment of hemocytes to the wasp egg. (A) Lamellocyte attachment to the wasp egg in the *Oregon-R* and in the (B) *ras*^*2*^ larvae. (C) Proportion of partially or completely encapsulated and melanized wasp eggs 72h after immune induction in the *ras*^*2*^ mutant or (D, E) after RNAi silencing. The numbers in parenthesis indicate the number of the examined *Drosophila* larvae. The error bars indicate the standard error of the mean **p<0.01, ***p<0.001. The scale bars indicate 20 μm.

We studied the morphological features of the capsules formed around the parasitoid egg by staining the capsule with the lamellocyte specific Atilla antibody. In *Oregon-R*, the hemocytes formed a tight, continuous sheet around the parasite egg (Fig 3A) with strong melanization masking the nuclear staining, while in the *ras*^*2*^ mutant the lamellocytes formed a loose network around the egg (Fig 3B), accompanied with marginal melanization 72h after infestation (Fig 3C). We obtained similar results after RNAi silencing by driving the two different RNAi lines with the *He-Gal4* driver line (Fig 3D and 3E), and with the lamellocyte specific *msnF9mo-Gal4* driver (S8 Fig).

### The number and morphological features of the circulating hemocytes in the *ras*^*2*^ mutant

**Fig 4:**
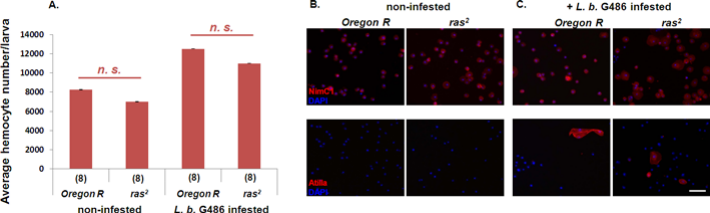
The number and morphology of circulating hemocytes in non-infested and immune induced *Oregon-R* and *ras*^*2*^ larvae. (A) Total hemocyte number of non-infested and *L*. *boulardi* G486 infested (72h) larvae originated from *Oregon-R* or *ras*^*2*^ mutant. n.s. means non-significant difference. (B) Hemocytes of non-infested or (C) *L*. *boulardi* G486 infested larvae (72h) were stained with plasmatocyte specific anti-NimC1 or with lamellocyte specific anti-Atilla antibody [38]. The scale bar indicates 20 μm.

Proliferation and differentiation are GTP-dependent processes in lymphocytes [41], and the IMPDH catalyzes the rate-limiting step of *de novo* synthesis of guanine nucleotides [24]. Therefore we tested whether the observed defect in capsule formation in the *ras*^*2*^ mutant could be the result of a decreased or abnormal hemocyte count in the hemolymph. Hemocyte counts of infested and non-infested *Oregon-R* and *ras*^*2*^ lines were determined, and the lamellocytes of the infested larvae were visualized. We found that the number of hemocytes (Fig 4A) was comparable and the number, as well as the morphological characteristics of circulating lamellocytes were the same (Fig 4B and 4C).

### *raspberry* is involved in the formation of pseudopod-like cytoplasmic extensions in hemocytes

**Fig 5:**
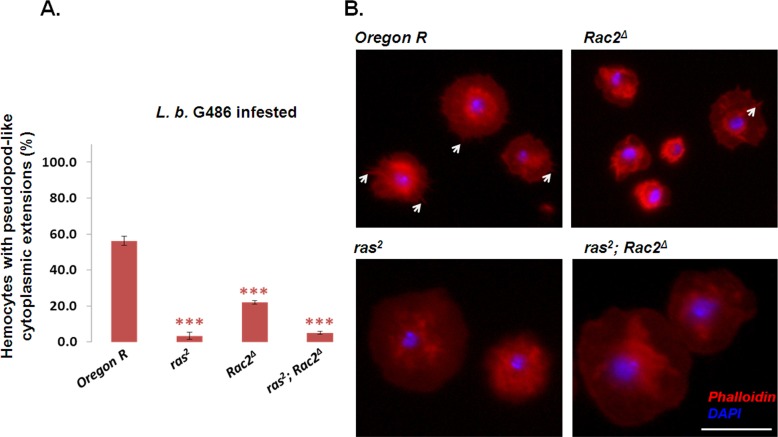
Formation of pseudopod-like cytoplasmic extensions in hemocytes. (A) The proportion of the filopodia-like extensions in hemocytes. (B) The morphology of circulating hemocytes in immune induced larvae 24 h after infestation. Arrowheads indicate the cytoplasmic extensions in hemocytes. The statistical analysis was performed counting at least 100 hemocytes derived from six larvae from each genotype. The experiment was repeated at least three times. The arrows show phalloidin-binding cytoplasmic extensions. The error bars indicate the standard error of the mean ***p<0.001. The scale bar indicates 20 μm.

After immune induction, hemocytes undergo functional and morphological changes. As *Rac2* has a role in these alterations [20], we tested *ras*^*2*^ mutants for morphological changes using *Oregon-R* and *Rac2*^*Δ*^ as controls. In the *ras*^*2*^ and *ras*^*2*^*; Rac2*^*Δ*^ larvae, hemocytes attached to the microscope slide were round with smooth margins and larger in size as compared to the *Oregon-R*, which had many pseudopod-like cytoplasmic extensions that show actin staining 24h after the immune induction (Fig 5). We found that the hemocytes of *Oregon-R* larvae had significantly more filopodia (7.2 ±2.9) compared to the *ras*^*2*^ (3.9±1.3) (p<0.01) and *ras*^*2*^*; Rac2*^*Δ*^ (4.3±2.1) (p<0.01), but this difference was not significant compared to the *Rac2*^*Δ*^ (5.6±4.0). In the *ras*^*2*^*; Rac2*^*Δ*^ double mutant, the proportion of the extension forming plasmatocytes was lower compared to the *Rac2*^*Δ*^ (Fig 5A), which suggests that *ras* is epistatic over *Rac2*. The length of the filopodia was similar in the *Oregon-R*, *ras*^*2*^, *Rac2*^*Δ*^ and *ras*^*2*^*; Rac2*^*Δ*^ (1–3 μm).

## Discussion

The hemocytes of *Drosophila melanogaster* larvae form tightly compact melanized capsules around parasitoid wasp eggs. This cellular encapsulation reaction has long been studied in *D*. *melanogaster* and in other *Drosophila* species [3, 6], however not much is known about the regulation of this defense mechanism. Small GTPases Rac1, Rac2 and the Jun N-terminal kinase Basket were found to be required for the proper encapsulation reaction against the endoparasitoid wasp *L*. *boulardi* [20, 21]. Additionally, the *Drosophila* βPS-integrin Myospheroid is necessary for hemocytes to adhere to the cellular capsule surrounding *L*. *boulardi* eggs, and Rac1 is required for the topographic localization of Mysopheroid in the cell membrane [42]. In a directed genetic screen, several genes were isolated that are related to encapsulation defects. The mammalian homologues of many of these genes are involved in wound healing, cellular adhesion and thrombosis [43].

In a screen [44] for genes involved in the regulation of cellular immune responses, we isolated a *raspberry* mutant (*ras*^*2*^) that shows significantly decreased chances for survival of the *Drosophila melanogaster* host after parasitisation with the parasitoid wasp *Leptopilina boulardi*. The *ras*^*2*^ mutant has a 5.0 kb insert in the *raspberry* gene [39]. We localized the site of the insertion in the second intron (data not shown). We found significantly higher number of capsules with loosely attached lamellocytes and improper melanization compared to the tight, compact and melanized capsules of the *Oregon-R* control. This shows that both the adhesion of lamellocytes and the melanization are affected by the mutation or, alternatively, improper lamellocyte attachment may inhibit melanization. To validate our results, we also used two independent *ras* RNAi constructs. We found that the two RNAi lines showed different efficiency, which may be due to differences in their genetic backgrounds. Both *ras* RNAi lines were able to mimic the mutant dark red eye color phenotype when driven with the *lozenge-Gal4* driver (data not shown), which indicated that the RNAi lines were fully functional. We noticed similar phenotypes after knocking down the *raspberry* transcript in hemocytes with the *msnF9mo-Gal4* and the *He-Gal4* drivers similarly to Bausek and Zeidler [45] after reduction of Ga73B levels in a *Hop*^*T42*^ background. The lower *Ga73B* gene dose, compared to the wild type pre-tumors, prevented the formation of the tightly bound cell mass and resulted in the formation of loosely associated cell clumps, which were not able to develop and generate the melanized tumors visible in adults. These defects were not associated with a decrease in hemocyte number or abnormal lamellocyte morphological features. Ga73B is a GTP binding protein, the α subunit of the heterotrimeric G proteins and a novel JAK/STAT pathway gene involved in the JAK/STAT-mediated tumor formation. The JAK/STAT pathway is also involved in the proper encapsulation response [46, 47].

The regulation of the actin-based membrane protrusions, such as lamellipodia and filopodia, require coordinated events in cytoskeletal remodeling. Central to this process are the small GTPases [8, 20, 48]. Incomplete encapsulation reactions, similar to those we have observed in the *ras*^*2*^ mutant, were described in the *Rac2*^*Δ*^ mutant, in which plasmatocytes and lamellocytes adhered to the parasitoid egg fail to spread and there is a failure of melanization [20]. Rac2 is a member of the Ras small GTPase superfamily involved in the formation of filopodia and lamellipodia [49, 50]. Filopodia formation of hemocytes in the *ras*^*2*^ and in the *Rac2*^*Δ*^ mutant was defective, suggesting that similarly to the *D*. *melanogaster Rac2* [20], *myospheroid* [42] and *TM9SF4* [51], the *Drosophila raspberry* gene is also involved in the encapsulation reaction via regulation of filopodia or lamellopodia formation. The lower proportion of filopodia forming plasmatocytes in the *ras*^*2*^*; Rac2*^*Δ*^ double mutant suggests that *ras*^*2*^ has an epistatic effect on *Rac2*^*Δ*^. Interestingly, it was shown that inhibition of the *de novo* GMP synthesis pathway has a strong effect on small GTPase function, including Rho GTPases such as Rac2 [52, 53]. Additional evidence for the involvement of *de novo* synthesis of guanine nucleotides in cellular processes was found in axon guidance too [54], where the Burgundy catalyzes the final reaction of the *de novo* GMP synthesis, while Raspberry catalyzes the first step of the same pathway both in neurons [54] and in hemocytes.

Possible explanation for the role of *raspberry* in the encapsulation reaction is that it encodes the rate-limiting enzyme for GTP synthesis and thus influences the function of enzymes requiring GTP. It is known that G proteins and small GTPases are involved in the regulation of processes related to the immune response. However, further studies must be conducted to elucidate the exact pathways affected in the case of decreased IMPDH level in the encapsulation reaction.

## Supporting Information

S1 Fig The eclosion of *D*. *melanogaster* versus *L*. *boulardi* G486 from RNAi silenced pupae. Two independent RNAi line were used (*y*^*1*^ sc* *v*^*1*^*; P{TRIP*.*HMC03250} attP2* and *y*^*1*^ *v*^*1*^*; P{TRIP*.*JF01446} attP2*) driven by *msnF9mo-Gal4* driver line. The eclosion rate was monitored at 25°C. The numbers in parenthesis indicate the number of the examined *D*. *melanogaster* pupae. The error bars indicate the standard error of the mean. *p<0.05, **p<0.01.
(TIF)

S2 Fig The eclosion of *D*. *melanogaster* versus *L*. *boulardi* G486 from RNAi silenced pupae. Two independent RNAi line were used (*y*^*1*^ sc* *v*^*1*^*; P{TRIP*.*HMC03250} attP2* and *y*^*1*^ *v*^*1*^*; P{TRIP*.*JF01446} attP2*) driven *He-Gal4* driver line. The eclosion rate was monitored at 25°C. The numbers in parenthesis indicate the number of the examined *D*. *melanogaster* pupae. The error bars indicate the standard error of the mean. *p<0.05.
(TIF)

S3 Fig The eclosion of *D*. *melanogaster* versus *L*. *boulardi* G486 from RNAi silenced pupae. Two independent RNAi line were used (*y*^*1*^ sc* *v*^*1*^*; P{TRIP*.*HMC03250} attP2* and *y*^*1*^ *v*^*1*^*; P{TRIP*.*JF01446} attP2*) driven *He-Gal4* driver line. The eclosion rate was monitored at 29°C. The numbers in parenthesis indicate the number of the examined *D*. *melanogaster* pupae. The error bars indicate the standard error of the mean. **p<0.01.
(TIF)

S4 Fig The proportion of living, partially or completely encapsulated and melanized wasp larvae. The encapsulation efficiency was examined at 48h following the wasp infestation. The numbers in parenthesis indicate the number of the examined *D*. *melanogaster* larvae. The error bars indicate the standard error of the mean.*p<0.05.
(TIF)

S5 Fig The proportion of living, partially or completely encapsulated and melanized wasp larvae in *raspberry* RNAi silenced *Drosophila* larvae. Two independent RNAi lines were used driven by *msnF9mo-Gal4*. The encapsulation efficiency was examined at 48h following the wasp infestation. The numbers in parenthesis indicate the number of the examined *D*. *melanogaster* larvae. The error bars indicate the standard error of the mean. **p<0.01, ***p<0.001.
(TIF)

S6 Fig The proportion of living, partially or completely encapsulated and melanized wasp larvae in *raspberry* RNAi silenced *Drosophila* larvae. Two independent RNAi lines were used driven by *He-Gal4*. The encapsulation efficiency was examined at 48h following the wasp infestation. The numbers in parenthesis indicate the number of the examined *D*. *melanogaster* larvae. The error bars indicate the standard error of the mean.*p<0.05, ***p<0.01, ***p<0.001.
(TIF)

S7 Fig The proportion of living, partially or completely encapsulated and melanized wasp larvae in *raspberry* RNAi silenced *Drosophila* larvae. Two independent RNAi lines were used driven by *He-Gal4*. The encapsulation efficiency was examined at 72h following the wasp infestation. The numbers in parenthesis indicate the number of the examined *D*. *melanogaster* larvae. The error bars indicate the standard error of the mean. **p<0.01, ***p<0.001.
(TIF)

S8 Fig The proportion of melanized wasp eggs with tight or loose capsule structure in *msnF9mo-Gal4/UAS-rasRNAi* larvae. The numbers in parenthesis indicate the number of the examined partially or completely encapsulated and melanized wasp eggs at 72h following the immune induction. **p<0.01.
(TIF)
