# Supplementary figures and images for: The raspberry Gene Is Involved in the Regulation of the Cellular Immune Response in Drosophila melanogaster
Source: PLoS One. 2016 Mar 4;11(3):e0150910. doi: 10.1371/journal.pone.0150910 (PMC4778902; doi:10.1371/journal.pone.0150910)

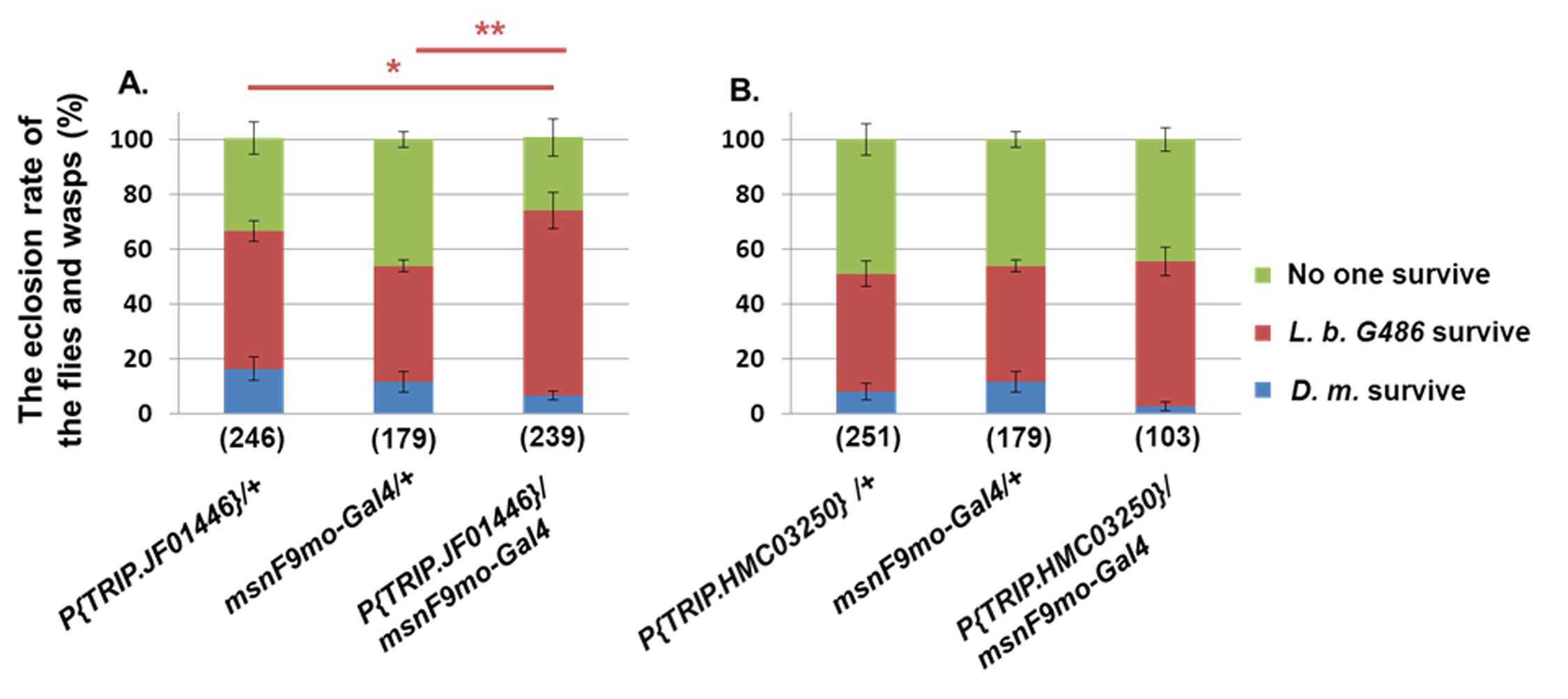

Supplement: S1 Fig — Two independent RNAi line were used (y1 sc* v1; P{TRIP.HMC03250} attP2 and y1 v1; P{TRIP.JF01446} attP2) driven by msnF9mo-Gal4 driver line. The eclosion rate was monitored at 25°C. The numbers in parenthesis indicate the number of the examined D. melanogaster pupae. The error bars indicate the standard error of the mean. *p<0.05, **p<0.01. (TIF) [file pone.0150910.s001.tif]

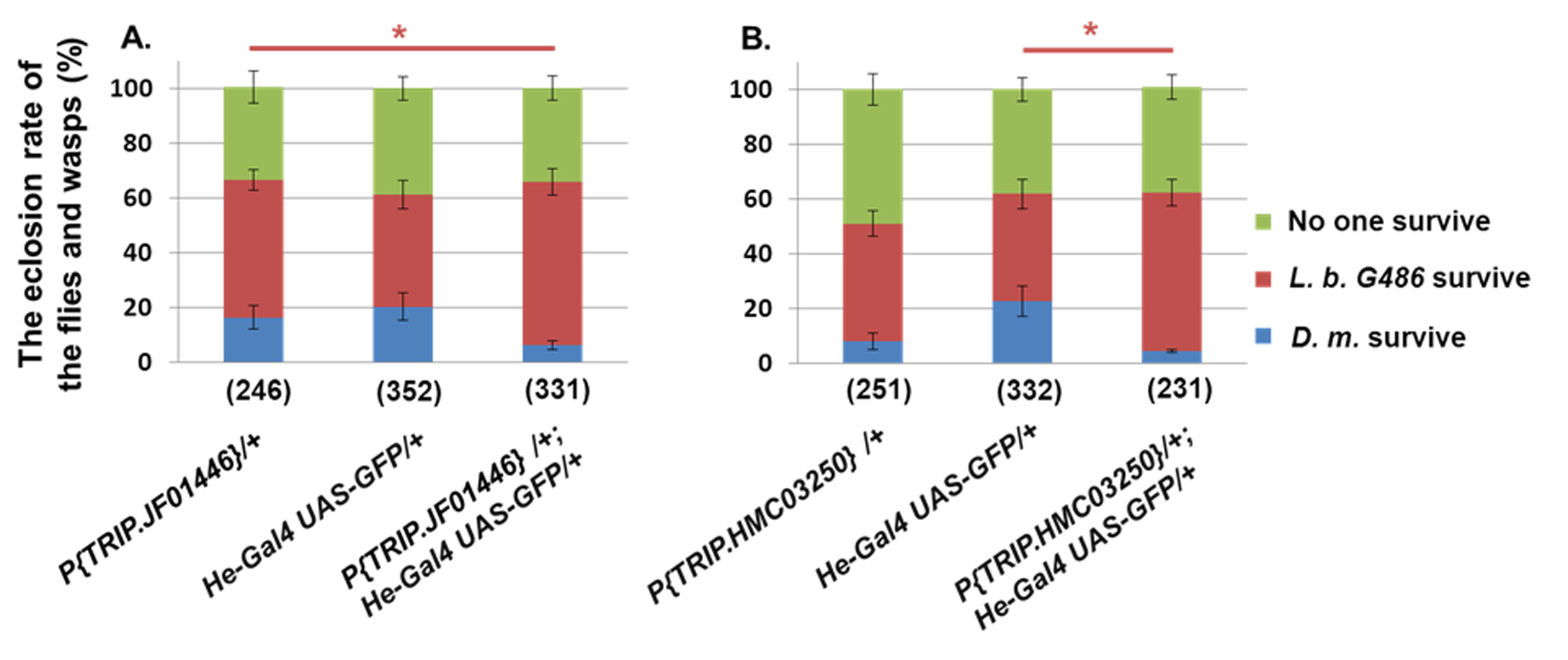

Supplement: S2 Fig — Two independent RNAi line were used (y1 sc* v1; P{TRIP.HMC03250} attP2 and y1 v1; P{TRIP.JF01446} attP2) driven He-Gal4 driver line. The eclosion rate was monitored at 25°C. The numbers in parenthesis indicate the number of the examined D. melanogaster pupae. The error bars indicate the standard error of the mean. *p<0.05. (TIF) [file pone.0150910.s002.tif]

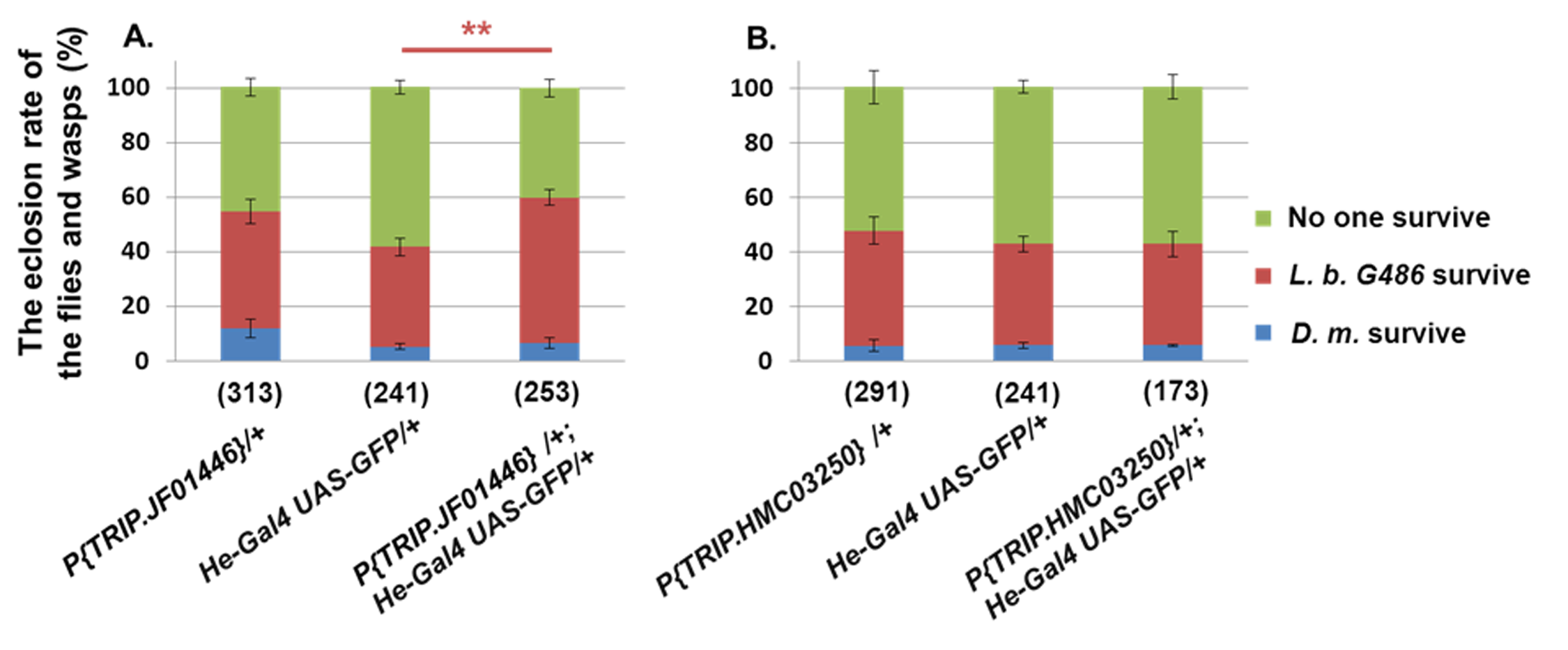

Supplement: S3 Fig — Two independent RNAi line were used (y1 sc* v1; P{TRIP.HMC03250} attP2 and y1 v1; P{TRIP.JF01446} attP2) driven He-Gal4 driver line. The eclosion rate was monitored at 29°C. The numbers in parenthesis indicate the number of the examined D. melanogaster pupae. The error bars indicate the standard error of the mean. **p<0.01. (TIF) [file pone.0150910.s003.tif]

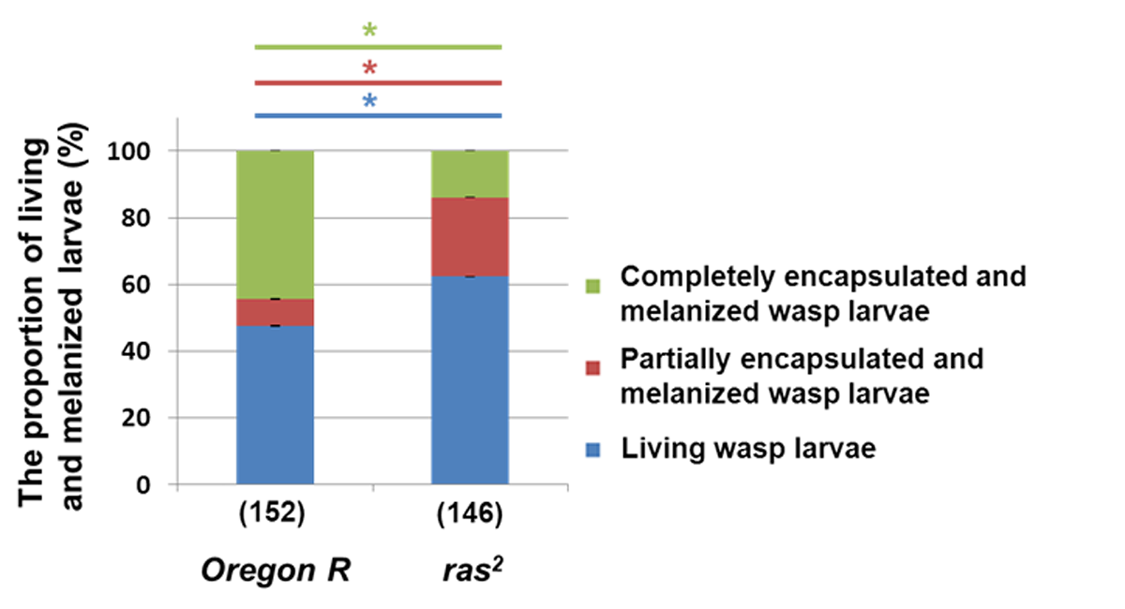

Supplement: S4 Fig — The encapsulation efficiency was examined at 48h following the wasp infestation. The numbers in parenthesis indicate the number of the examined D. melanogaster larvae. The error bars indicate the standard error of the mean.*p<0.05. (TIF) [file pone.0150910.s004.tif]

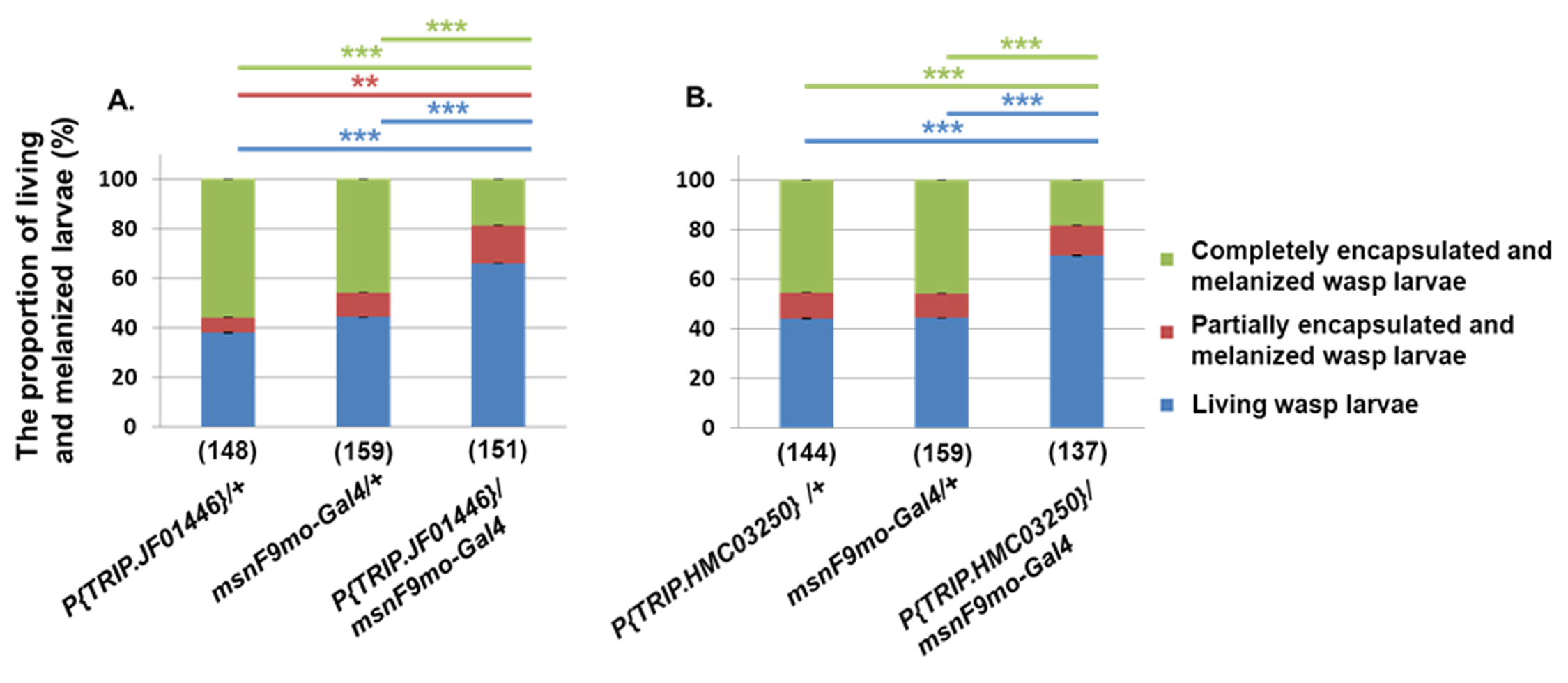

Supplement: S5 Fig — Two independent RNAi lines were used driven by msnF9mo-Gal4. The encapsulation efficiency was examined at 48h following the wasp infestation. The numbers in parenthesis indicate the number of the examined D. melanogaster larvae. The error bars indicate the standard error of the mean. **p<0.01, ***p<0.001. (TIF) [file pone.0150910.s005.tif]

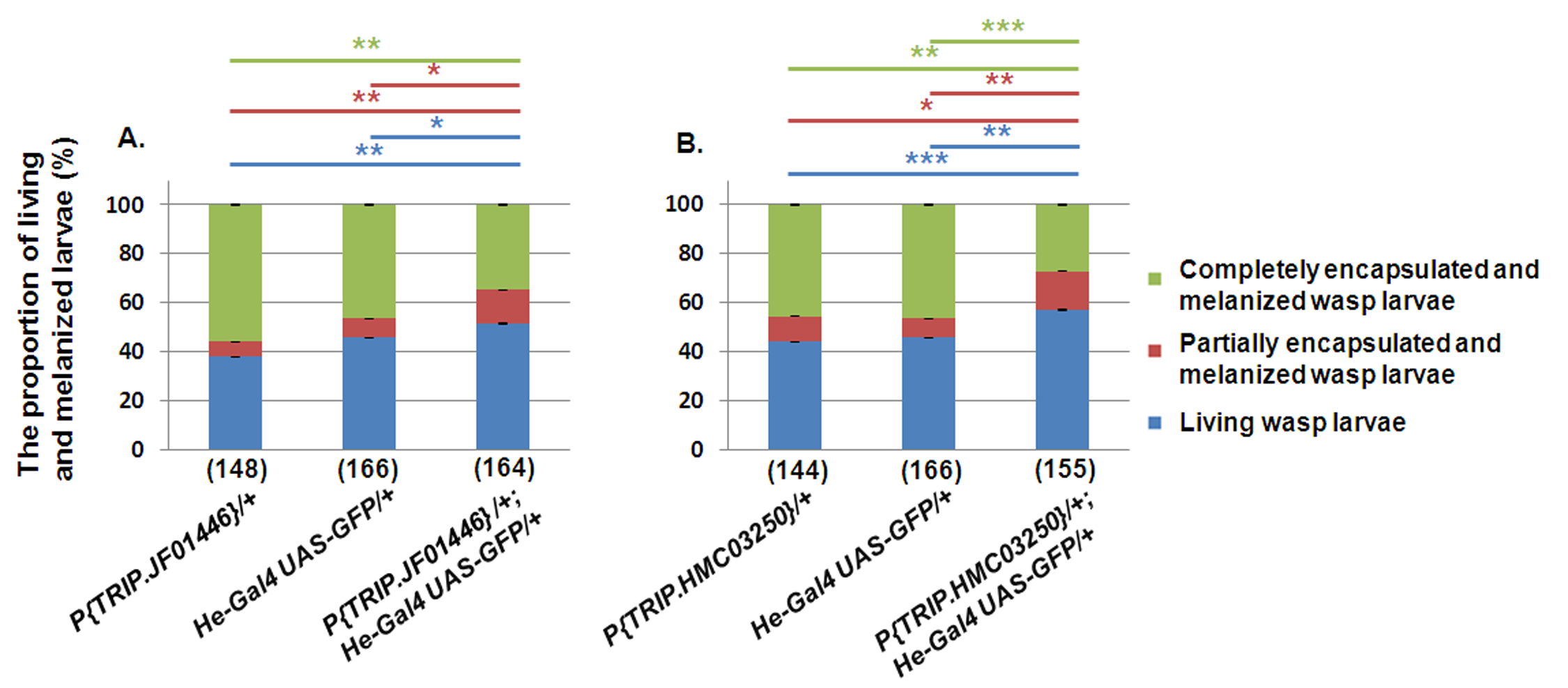

Supplement: S6 Fig — Two independent RNAi lines were used driven by He-Gal4. The encapsulation efficiency was examined at 48h following the wasp infestation. The numbers in parenthesis indicate the number of the examined D. melanogaster larvae. The error bars indicate the standard error of the mean.*p<0.05, ***p<0.01, ***p<0.001. (TIF) [file pone.0150910.s006.tif]

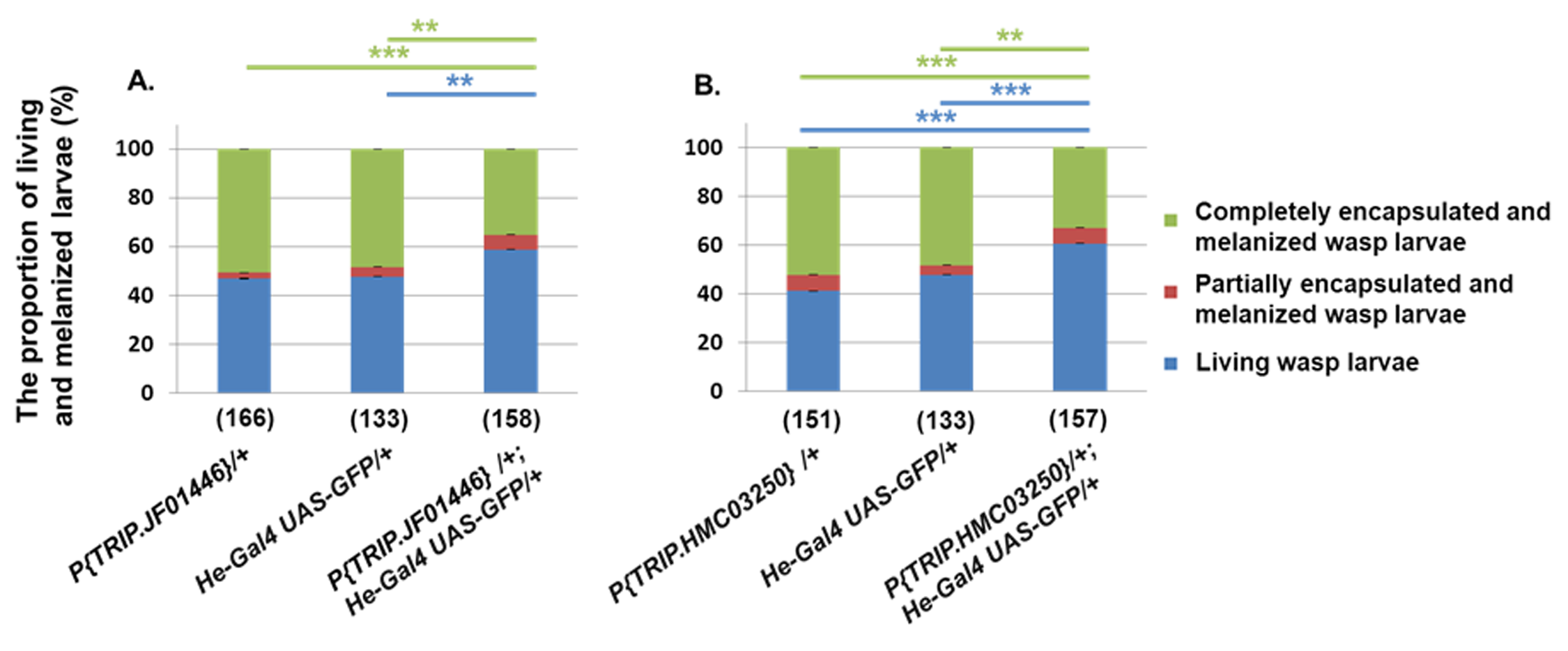

Supplement: S7 Fig — Two independent RNAi lines were used driven by He-Gal4. The encapsulation efficiency was examined at 72h following the wasp infestation. The numbers in parenthesis indicate the number of the examined D. melanogaster larvae. The error bars indicate the standard error of the mean. **p<0.01, ***p<0.001. (TIF) [file pone.0150910.s007.tif]

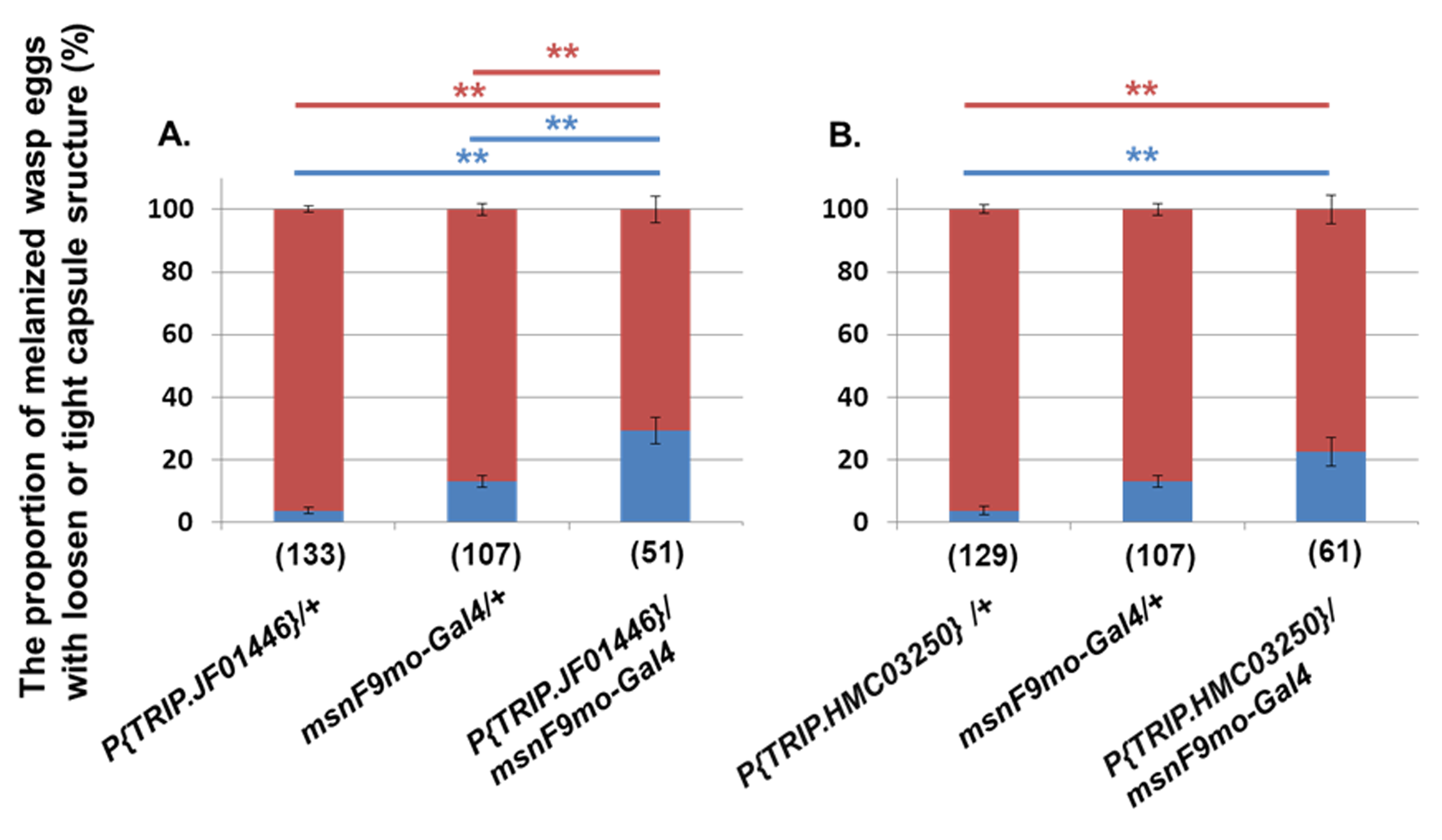

Supplement: S8 Fig — The numbers in parenthesis indicate the number of the examined partially or completely encapsulated and melanized wasp eggs at 72h following the immune induction. **p<0.01. (TIF) [file pone.0150910.s008.tif]
